# Use and Effectiveness of Social-Media-Delivered Weight Loss Interventions among Teenagers and Young Adults: A Systematic Review

**DOI:** 10.3390/ijerph18168493

**Published:** 2021-08-11

**Authors:** Blanca Lozano-Chacon, Victor Suarez-Lledo, Javier Alvarez-Galvez

**Affiliations:** 1 Computational Social Science DataLab (CS2 DataLab), University Institute for Social Sustainable Development (INDESS), University of Cadiz, 11003 Cádiz, Spain; blanca.locha90@gmail.com (B.L.-C.); victor.sanz@uca.es (V.S.-L.); 2 Department of Biomedicine, Biotechnology, and Public Health, University of Cadiz, Avda. Ana de Viya, 52, 11009 Cádiz, Spain; 3 Institute of Research and Innovation in Biomedical Research of Cadiz (INIBICA), University of Cadiz, 11405 Jerez de la Frontera, Spain

**Keywords:** social media, interventions, weight loss, teenagers, young adults

## Abstract

Obesity is a risk factor that exponentially increases morbidity and mortality in the world. Today, new health strategies are being implemented based on the use of social media but the use and effectiveness for these interventions needs to be assessed. The objective of this systematic review is to assess the impact of social-media-delivered weight loss interventions among teenagers and young adults. We searched PubMed, Scopus, Google Scholar, PsycINFO, and OVID to identify articles that focused on this topic. Fourteen studies were included in the final review. The commitment of the participants was found to be fundamental factor when assessing the impact of social-media-delivered weight loss interventions, but also the social context in which the interventions were carried out. Our study highlights the potential of social media platforms to address weight loss interventions among younger groups. The works evaluated showed the usefulness of social media for the adequate monitoring and control in these groups. Finally, the current variety of study designs in this field highlights the need for greater homogeneity in their methodology and applications, which is a fundamental step before these tools could be considered a suitable tool for overweight management in clinical practice.

## 1. Introduction

Obesity and overweight are major health concerns in today’s societies, accounting for a significant proportion of the global burden of disease [1,2]. During recent decades, obesity has reached epidemic proportions in developed countries. In fact, obesity has become one of the main causes of morbidity and mortality in the world. More than 1.9 billion adults (aged 18 years or older) were found to be overweight, of which more than 650 million were obese, in 2016 [3]. Obesity and overweight are risk factors that increase morbidity and mortality due to the risk of developing various pathologies, mainly strokes and diabetes, but also other acute and chronic diseases such as cancer, osteoarthritis, liver and kidney disease, and sleep apnea [4]. Obesity has been found to be associated with depression, poor quality of life and poor levels of social and psychological well-being across the lifespan [2,5].

Different communicative strategies have been developed and implemented to prevent obesity [6]. These include advertising campaigns through radio and/or television, educational information through newspapers, text messages and emails, and educational programmers in primary care and schools, internet campaigns, video games and, very recently, social media such as Twitter or Facebook [7,8]. In recent years, many studies have identified social media as a promising instrument in fighting overweight and obesity among population [9]. Social media presents significant opportunities that could positively affect public health through the propagation of information that could prevent unhealthy habits linked to obesity (i.e., sedentary lifestyles, high-fat diets, etc.). These tools can easily be used to spread evidence-based health-related knowledge online regarding healthy habits and behaviors which promote health among specific social groups that can share information about healthy diets and behaviors. 

Consequently, the possibilities for combating obesity and overweight through digital tools have increased in the last years [10]. Likewise, social media platforms have increased the public availability of health data regarding opinions, attitudes, behaviors, and experiences of users, and this knowledge can also be useful for identifying healthy habits and preventing risky behaviors related to overweight and obesity [11]. Therefore, an appropriate use of social media could contribute to a greater acquisition of medical skills among the population, self-efficacy in fulfilling treatments, adherence in therapies, and disease prevention [12]. Recent evidence supports the use of social media platforms to increase health promotion interventions, by providing social support and guidance to those undergoing health behavior changes such as those related to weight loss (i.e., mostly through physical activity or healthy diets) [13].

However, although different studies assume the advantages and possibilities of social media to combat overweight and obesity, the effects of health programmers and interventions for weight-management through social media still remain unclear [9]. Despite this, we can find some initial studies that have tried to answer this question but it is unknown to what degree social media can prevent and control obesity and, in particular, through interventions that are not exclusively based on dietary changes [14]. Therefore, the role of social media in online weight management remains empirically unclear [13]. In particular, recent studies have found difficulties in comparing the effects of interventions and programmers applied to different social groups (young people, adults, students, patients, etc.) and using diverse online applications [15]. Thus, it is difficult to determine the real usefulness and impact of these platforms for public health [13,16,17,18,19,20,21,22,23,24,25,26,27,28,29]. 

In this study, we start from the premise that the fundamental problem in answering this question is mainly found in the population diversity of the studies carried out. For this reason, in this study we focus on the young population (i.e., adolescents and young adults) since, according to current evidence, we know that at this stage of our life cycle fundamental changes occur at the physiological, emotional and behavioral levels that will condition our future body weight [30]. In fact, we know that the years after puberty are essential to understand overweight and obesity [31], as early adolescent changes in body weight may cause emotional and behavioral responses that frequently include efforts for overweight management [32]. In parallel, other changes in young populations’ lifestyle related to healthy habits (e.g., miracle diets and decreasing physical activity) and high-risk behaviors (e.g., tobacco, alcohol and drug consumption) have the potential to affect weight [33]. Furthermore, teenagers and young adults (TYA) are the age groups that present the highest use and exposition to online health-related contents [34], and in particular through social media platforms such as Facebook, Twitter, Instagram or YouTube.

In order to overcome these problems, the objective of this systematic review is to assess the use and effectiveness of social-media-delivered weight loss interventions among TYA. Trying to shed light in this new research field, we focus here exclusively on young social media users, i.e., a homogeneous group that present similar interests, and a higher use, and general exposition to these platforms [34]. 

## 2. Materials and Methods

This systematic review was carried out in accordance with the Preferred Reporting Items for Systematic Reviews and Meta-Analysis (PRISMA) protocol for systematic reviews [35]. The PRISMA checklist was carefully followed in the process of developing this research. 

### 2.1. Data Sources and Search Terms

We searched Pubmed, Scopus, Google Scholar, Psycinfo, OVID, and EMBASE—the main search engines in health sciences—for articles that studied the effect of social-media-delivered weight loss interventions among TYA. Due to the relatively recent appearance of social media (e.g., the term social media was not incorporated as a Medical Subject Heading–MeSH until 2012), the search was restricted to research papers produced from 2010 to 2021. The last update was conducted on 6 March 2019. Our research terms focused on three basic dimensions: (a) social media (‘social media’ OR ‘online social network’ OR ‘online community’ OR ‘YouTube’ OR ‘Facebook’ OR ‘Twitter’ OR ‘Instagram’ OR ‘Myspace’ OR ‘Flickr’), AND (b) weight management (‘obesity’ OR ‘overweight’ OR ‘bmi’ OR ‘body mass index’ OR ‘weight gain’ OR ‘weight loss’ OR ‘diet’ OR ‘physical activity’), AND (c) age group (‘adolescents’ OR ‘teenagers’ OR ‘young adults’ OR ‘young’). Full details of search terms and Boolean combinations are included in the Appendix A.

### 2.2. Inclusion and Exclusion Criteria

Only empirical research articles and reviews written in English and Spanish which focused on the use of social-media-delivered weight loss interventions among TYA adults were selected. In particular, our review included papers focusing on young people aged 13–35 years that were published during the period 2010–2021. Taking into account that social media has only recently been included in interventions for weight management, we considered research evidence from different study designs: descriptive studies using survey methods, longitudinal studies, systematic reviews and meta-analysis, pilot studies, randomized controlled trials, qualitative, and mixed methods studies. Abstracts, doctoral theses, magazines, editorials, press articles, comments, letters, book reviews, reports and other documents which did not meet the inclusion criteria were excluded.

### 2.3. Study Selection

After conducting the searches in the different databases, the authors began the inclusion/exclusion process according to the eligibility criteria. First, those articles that did not comply with previously defined eligibility criteria were eliminated. Second, titles and abstracts were systematically screened according to the inclusion/exclusion criteria. Finally, the full-text articles were retrieved and fully assessed according both their methodological quality and their global adjustment to the eligibility criteria before being selected in the review. The reliability of the study selection process was assessed by two of the authors (B.L.-C. and J.A.-G.) Cronbach’s Alpha. The inter-reviewer selection showed high inter-rater reliability (α = 0.92). Between the 80 articles fully assessed, only three discrepancies were found and lately resolved by mutual agreement with a third coauthor (V.S.-L.). Finally, Mendeley Desktop was used to manage each of the documents so that they were stored in a database.

From every article, we selected the following data: author(s), year of publication, population, country, objectives, social media platform (Facebook, Twitter, Instagram, YouTube, etc.), study design, measured variables (weight, height, body mass index, obesity, physical activity, healthy habits, etc.), and main results. 

### 2.4. Risk of Bias in Individual Studies

In order to reduce the risk of bias and increase the final quality of our selection, the studies were assessed and critically appraised with a scoring system of ten questions to assess the methodological quality of the articles included [36]: (Q1) Did the study address a clearly focused issue?; (Q2) Did the authors use appropriate methodology to answer their question?; (Q3) Was the study population clearly specified and defined?; (Q4) Were measures taken to accurately reduce measurement bias?; (Q5) Were the study data collected in a way that addressed the subject of the research?; (Q6) Did the study have enough participants to minimize speculation?; (Q7) Did the authors take sufficient steps to ensure quality in the study data?; (Q8) Was the data analysis sufficiently rigorous?; (Q9) How complete is the discussion?; (Q10) To what extent can the results be generalized to other international contexts? Each of these questions were categorized according to the following categories: ‘poor’ (score = 1), ‘fair’ (score = 2), ‘good’ (score = 3), and ‘unable to say’ (score = 0). We calculate the average quality assessment score for each study and the percentage of the total results. 

In addition, we also rated the quality of the studies using the method developed by Norman et al. [37]. This tool was selected because it was particularly designed to evaluate the general methodological quality of online interventions. Therefore, we found it appropriate to complement the previous quality assessment scale with the scoring system designed by Norman, since our main objective was to study the use and effectiveness of social media on self-management of weight loss. The Norman score was based on nine characteristics that include the evaluation of: (Q1) randomization; (Q2) the inclusion of a control group; (Q3) technology isolation; (Q4) the pre-test design; (Q5) retention; (Q6) the equivalence of the reference group; (Q7) missing data; (Q8) calculations of the sample size and (Q9) the validity of the outcome measures. For each criterion present, the study received one point, with a maximum score of nine. 

In these scoring systems, items with a value above 66% were considered to be high-quality items, those with a value between 66% and 33% would be considered of moderate quality and items with a value below 33% would be considered to be low quality (i.e., including those that did not provide enough information to assess their quality). Finally, by combining the results of these tools, we calculated the average percentage to develop a rating of the studies with the higher methodological quality. 

## 3. Results

### 3.1. Description of the Studies

**Figure 1:**
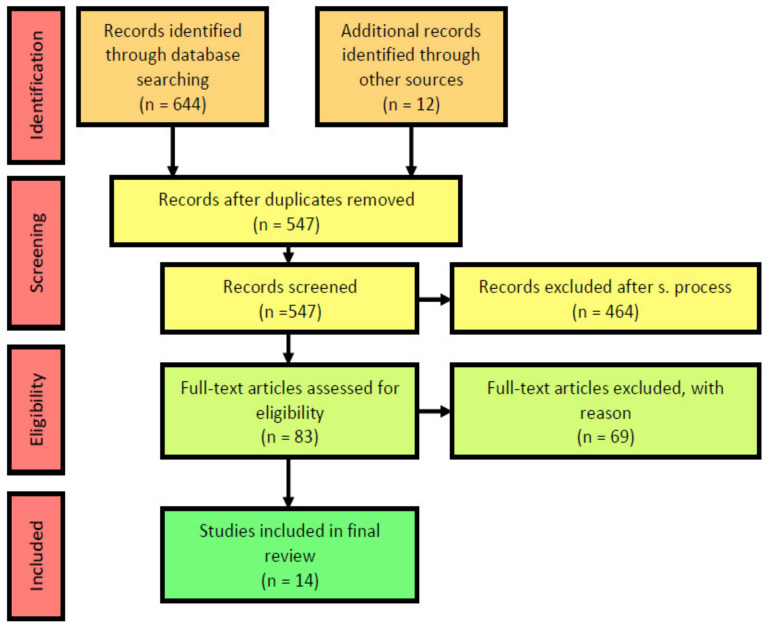
PRISMA flow diagram.

After performing the searches in each of the aforementioned databases, a total of 656 articles were identified (i.e., 644 were extracted from the abovementioned data sources, and 12 articles were found from other research articles), of which 109 were duplicates. Of the remaining 547, 464 were excluded after examining the title and abstract for not meeting the eligibility criteria (i.e., excluded after screening process), leaving a total of 83. After a full-text reading, 14 articles met the inclusion criteria (Figure 1).

### 3.2. The Use of Social Media for Weight Loss Interventions

Most of the weight loss interventions were implemented through Facebook (10/14). These articles were published between 2012 and 2021. The studies were conducted in different parts of the world but were predominantly in the United States: Australia (*n* = 1), Canada (*n* = 1), Egypt (*n* = 1), Germany (*n* = 1), India (*n* = 1), Japan (*n* = 1), and United States (*n* = 8). Nine analyzed weight loss through physical activity and healthy lifestyle habits [38,39,40,41,42,43,44,45,46], two articles were focused on physical activity [47,48] and three articles studied weight loss but also mentioned the effect on mental health [49,50,51]. The types of studies included were randomized controlled trial (*n* = 6), experimental design (*n* = 3), online survey (2), controlled-trial nonrandomized (*n* = 2), and content analysis (*n* = 1) that were mainly aimed to measure body mass index (BMI), eating habits, physical activity and their relationships (10/14). Additionally, some of the studies included social, psychological, and behavioral measures such as social support, psychological functioning, depression, anxiety, perfectionism, impulsiveness, self-efficacy, commitment or motivation (7/14). The characteristics and main findings of the studies are described in Table 1.

#### 3.2.1. Diet and Eating Habits 

Although not all social media delivered interventions based on changes in diet or eating habits were successful in terms of weight loss, we found evidence that the use of these interventions can have a positive contribution in terms of participants’ self-management of weight and control. For instance, a nonrandomized controlled trial conducted among medical students observed a modest reduction in body mass index (BMI) which seemed to be due to the decrease in the intake of junk food. However, there were significant changes in fruit and vegetable intake, as well as in the practice of physical activity [41]. In addition, there was found the possibility of improving eating habits with other means, and not only with diets directed to the target population. For example, in the pilot study of Ling et al. [39], a group of young caregivers with children between 3 and 5 years of age were instructed in healthy eating habits. Thus, indirectly, the diet of the whole family was also improved as healthy cooking was encouraged and they were taught to read food labels to know and understand what they were really eating.

#### 3.2.2. Exercise and Physical Activity

On the other hand, the usefulness of social media to carry out interventions aimed at weight loss through the practice of physical activity was also highlighted. Pagkalos et al. [48], through social networking applications (SNApps) on Facebook, measured how participants performed physical exercise classified in two categories, low or moderate, and monitored the self-reports of each of the participants. These self-reports were compared with a pedometer to know the veracity of the self-reports and, finally, they showed that the use of applications through Facebook could be an innovative and powerful instrument for collecting and dynamically monitoring health data. Additionally, in a randomized controlled trial included in our systematic review, after carrying out an intervention by Facebook in order to guide the participants in a healthy lifestyle through the promotion of physical activity, results were considered unfavorable since participants did not effectively reduce BMI or increase their physical activity [47]. However, this intervention could be also considered positive from the point of view of health literacy promotion. In other words, although a change in habits did not take place, health knowledge was transmitted that could be applicable in future changes in lifestyles.

#### 3.2.3. Mental Health and Risk Behaviors 

Some of the interventions also considered the emotional and mental health of the participants as a way of understanding healthy behaviors and/or habits related to weight loss [49,50]. The cross-sectional study of Ramalho et al. [49], whose purpose was to determine the impact and compliance of an intervention delivered by Facebook for weight loss in the Portuguese population, revealed that the female participants had higher scores than the males in psychological disorders, unbalanced eating behaviors, impulsivity, were less active in school, and attained lower scores in health-related quality of life. In short, a set of differences that could determine the viability and success of the interventions carried out. According to Walker et al. [50] the excessive use of Facebook could also produce unexpected effects in terms of disorderly eating behavior (e.g., through online communities that might promote miracle diets or even unhealthy behaviors for rapid weight loss).

### 3.3. The Effectiveness of Social-Media-Delivered Weight Loss Interventions

Although by considering different independent variables and different study designs we cannot establish an overall measurement of the size of the effect produced by each of the studies, the vast majority of the interventions were considered effective in one way or another, either because: (1) they really managed to favor weight loss [21,42,43,44,46,51]; or, at least, (2) they promoted some healthy attitude or habit (for example, through improvements in diet, increased physical activity, greater commitment to change habits, increased health literacy and self-efficacy) [41,45,50]; or (3) because they made possible an improvement in the measurement systems, in the data collection and monitoring processes and/or in the control of the intervened population [21,39,40,45,48]. 

The effectiveness of the weight loss interventions was found to be associated with individual factors such as socioeconomic status (SES) [39], but also so with contextual factors such as the social support received and the personal social network (i.e., family members, friends, etc.) that could either reinforce or reduce the impact of the intervention. Factors that, according to Walker et al. [50], would be decisive in establishing the participant’s commitment throughout the intervention and that consequently may determine its success. For instance, Chang et al. [9] stressed that those participants whose social networks were diminished (i.e., those with a lower number of social contacts), would be more likely to follow social media delivered interventions and subsequently have a lower drop-out rate. In the same vein, as suggested by Napolitano et al. [38], participation in social media interventions might be beneficial for the more socially isolated individuals, i.e., those that might need some kind of reinforcement to start or maintain healthy behaviors.

Finally, some differences were found between social media delivered weight loss interventions and those based on traditional methods (i.e., brochures and questionnaires). With the former, a more significant weight loss was achieved than the latter even though both followed identical guidelines of diet and physical activity [38]. Another important aspect to note was the role social media played in health promotion, since those overcomes geographical and social barriers and can reach large groups of people anywhere while allowing a large number of cases to be closely traced and controlled in less time [41]. Furthermore, as observed, due to the inherent interactivity of these social platforms, young population may develop additional interest and critical thinking regarding their eating and physical activity habits. Consequently, this approach seems to reinforce mutual social support and peer education for youth health promotion [45]. 

Specifically, Facebook was found to be useful to share evidence-based weight loss intervention content (e.g., advice or warnings). This tool also offered additional advantages for interventions such as scheduled delivery of content, ease for users to stay in touch with their network and their respective coaches (i.e., intervention providers), and receive feedback from other participants. However, it was also demonstrated that the engagement and general interest of the platform users might easily decline over time [51]. Another disadvantage mentioned was that the use of this medium for weight monitoring is still underdeveloped and, thus, much remains to be discovered. One of the selected studies pointed to the need for more attention and control in the implementation of behavior change strategies through these open social platforms [46]. Therefore, in certain aspects much more research is needed to increase the comparability and improve the impact assessment of social media delivered weight loss interventions [39]. 

### 3.4. Methodological Quality of the Studies

**Figure 2:**
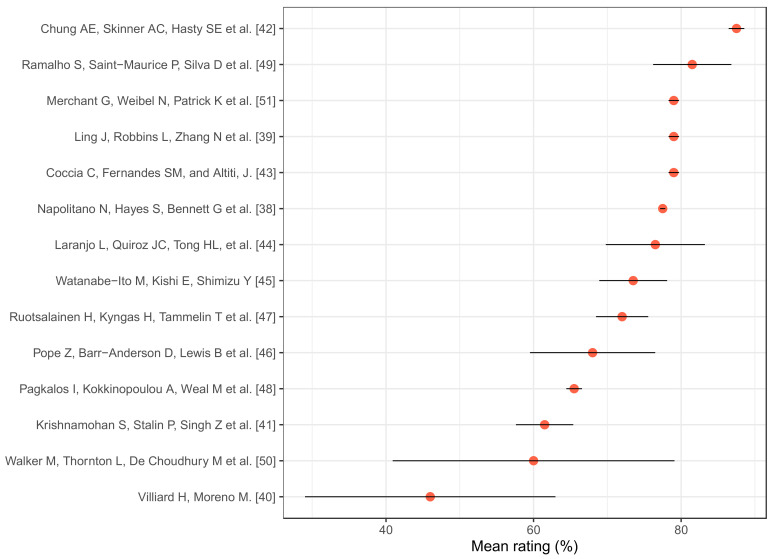
Methodological quality of selected studies measured by average percentage and standard deviations of the scores [38,39,40,41,42,43,44,45,46,47,48,49,50,51].

Studies that were selected, according to the first scoring system for the methodological quality assessment of the included studies, 13 articles were considered to be high quality with values above 66% and a just one article presented a moderate quality, with 64% (see Table A1 in the Appendix A). On the other hand, according to the scale developed by Norman et al. [37], 11 articles were of high quality with values greater than 66%, and the rest were of moderate (3) or low quality (1) (see Table A2 in the Appendix A). Finally, we calculated the average percentage between these two instruments in order to develop a rating of the studies with the higher methodological quality and lower risk of bias (Figure 2).

## 4. Discussion

To the best of our knowledge, this is the first article that specifically assesses the use and effectiveness of social-media-delivered weight loss interventions among TYA, a population group that despite having a high penetration on these platforms has not been studied in depth. 

Our findings show that social-media-delivered weight loss interventions are being progressively used among young populations. However, although the effectiveness of the interventions through the social media to lose weight was not evident in all the studies we found, the works evaluated showed the usefulness of these new tools for the adequate monitoring and control of the population on which the intervention is applied. That is to say, unlike classic interventions, these new tools make possible a better traceability and control of the target population and also their contacts (i.e., family members and friends). The possibility of mapping the social network of friends offers the advantage of understanding influences between peer groups, while we can observe and empirically measure mechanisms of social reinforcement and contagion that may ultimately determine the commitment to successfully carry out an intervention [50]. In any case, social media also appear to be a very useful tool for reaching individuals who may be socially isolated and who may be more reluctant to participate in face-to-face interventions [38].

On the other hand, in addition to facilitating the recording of participants’ data, since most of the procedures could be automated by means of online questionnaires and customized applications, this new type of weight loss intervention has the advantage of being able to capture the structure of the interaction network within the platform. In short, network data can be fundamental to understanding the mechanisms described above [48].

The interventions identified were not only based on the idea of following a diet but also to educating participants in healthy eating habits [39,40,47,48,52]. The advantages found were essentially the reduction of BMI, body weight and waist circumference in addition to the increase in physical activity. These mixed interventions indirectly helped to users reducing their exposure to cardiovascular risk factors [38,40,52,53,54,55,56,57]. It should be noted that social media manages to overcome geographical and social barriers, and this, when added to the significant use and impact these tools have in our society, results in the benefit of being able to reach large groups wherever they are and control numerous cases in a short time. Another advantage is the fact that social media are a great platform for health promotion and disease prevention [53].

In particular, we discovered that the effectiveness of the intervention depended on a combination of individual and contextual determinants. For instance, those users with a high SES, higher social support, and/or smaller social network (i.e., those with less face-to-face social relationships) showed a higher participation and commitment through social media platforms, and thus they commonly had a lower abandonment rate [9,58,59]. In addition, we found that social-media-delivered weight loss interventions were not only a benefit for the target groups, but also for other social media users too due to the relationships formed online which seem to be a valuable source of social support among young population with high level of digital health literacy [40,54,57].

However, despite all the benefits mentioned above, some inconveniences and contradictions remain evident. Not all studies detect significant weight loss or an increase in physical activity [41,47]. In addition, in the randomized controlled trial of Jane et al. [52], weight data and metabolic results were collected from self-report instruments so they may have introduced some bias in these results. Therefore, it is in part unclear whether this new type of intervention is truly useful among TYA. In fact, the result of social-media-delivered weight loss interventions has been found to be ambivalent and–in some cases–contradictory, which is probably due to the high variability of study designs and the different variables measured (BMI, body fat, physical activity, diet, eating habits, emotional and mental health outcomes, among other). 

We also found that our state of general well-being and quality of life was influenced by the intensity of social media use in an inverse way, that is, the greater the use, the lower our well-being, hence the relationship between the excessive use of Facebook, for example, with disorderly eating behaviors. In addition, the relationship between overweight and psychological disorders such as low self-satisfaction and loss of identity were found to be higher among young female social media users [49,50,51,60].

### Limitations and Implications of the Findings

We found some limitations when conducting our systematic review. On the one hand, due to the novelty of the use of social media as a tool for delivering weight loss interventions, we detected significant heterogeneity in the results in the articles included which has made it difficult for us to compare them and measure the overall impact in weight loss. Moreover, considering the rapid evolution and inherent dynamism of the social media ecosystem, in the near future it would be necessary to incorporate new online platforms that could be interesting from the point of view of implementing weight loss interventions. On the other hand, our review was limited to articles in English and Spanish, which in part limits the generalization of the findings to English and Spanish-speaking countries. 

In any case, the works reviewed showed indirect benefits such as the development of healthy habits and the increase in health literacy among young population (knowledge and behaviors that can be shared and extended through mechanism of positive social contagion), as well as the usefulness of these new tools for the adequate monitoring and control of the population on which the intervention is applied.

## 5. Conclusions

Social media are becoming a basic tool in the daily lives of a wide section of society which means that it would be interesting to continue studying its effectiveness and adequacy for addressing obesity and overweight among the younger groups of our societies. In our review, we have detected different factors that are relevant when seeking to strengthen the commitment of users to follow certain medical guidelines. We have therefore shown that tailored health interventions will be fundamental in supporting weight loss among diverse socio-demographic groups. In addition, the social context in which the interventions are carried out must also be considered. On the other hand, the findings of this systematic review demonstrate the importance of taking into account the impact of social-media-delivered interventions on the mental health and risk behaviors of users and the fact that an intervention that could be beneficial in principle may actually have unwanted negative consequences caused by the mediated effect of the social platform we use.

Finally, given that there are clear benefits but also some disadvantages to using social media for weight self-management, it would be interesting to pursue future studies aimed to increase comparability with existing evidence.

## Figures and Tables

**Table 1:** Characteristics of studies included in the review, objectives and main findings.

Author	Year	Population	Country	Objectives	Social Media	Study Design	Variables	Main Results
**Napolitano N, Hayes S, Bennett G et al.** [38]	2013	University students between 18–23 years old	USA	Examine the feasibility, acceptability and initial efficacy of a technology-based weight loss intervention.	Facebook	Randomized controlled trial	BMI, physical activity, self-efficacy, weight self-efficacy, social support, commitment, compliance	The data indicated that a combination of Facebook plus SMS text messages, comments and identification of a support person produced significantly greater weight losses than Facebook alone or a waiting list control group.
**Ling J, Robbins L, Zhang N et al.** [39]	2018	Children between 2–5 years old, young caregivers with a mean age of 28.7 years	USA	Examine the viability and preliminary effectiveness of using Facebook in a 10-week lifestyle intervention with head-start caregivers and preschoolers to improve healthy behaviors and reduce body mass index.	Facebook	Randomized controlled trial	BMI, waist circumference, eating habits, physical activity	The prevalence rate of overweight and obesity (46.3%) in this study is approximately 1.33 times higher than the reported national prevalence rate of 34.9% among Head Start preschoolers (Aikens et al., 2011). Similarly, the prevalence rate of overweight and obesity (81.2%) among caregivers is also much higher than the 60.4% prevalence rate reported among Michigan women (Kaiser Family Foundation, 2017). All these statistics highlight the critical need to target low-income Head Start families to help them achieve a healthy weight.
**Villiard H, Moreno M.** [40]	2012	Young adults between 18–20 years old	USA	Determine (a) how and to what extent college students are discussing physical fitness on Facebook, and (b) how user-generated fitness information is linked to product announcements and fitness tips.	Facebook	Content analysis	Eating habits, physical activity, weight concerns, body image	Our findings illustrate that physical fitness is a common topic on Facebook. Overall, more than 70 percent of the profiles evaluated referred to physical fitness behaviors on Facebook, and most referred to physical activity. The findings also illustrate that physical fitness, specifically, is a topic on which Facebook recognizes and makes ads by linking specific key words or phrases within the status updates generated.
**Krishnamohan S, Stalin P, Singh Z et al.** [41]	2017	Young adults between 18–23 years old	India	Measure the effectiveness of health education using social networking sites to promote a healthy lifestyle among medical students in Puducherry, India	Facebook	Nonrandomized controlled trial	Physical activity, healthy eating, BMI	In general, it was found that the use of Facebook did not significantly improve the consumption of healthy diet and physical activity among medical students, except in reducing the consumption of junk food.
**Chung AE, Skinner AC, Hasty SE et al.** [42]	2020	Young adults between 19–20	USA	We developed and pilot tested a mHealth intervention, “Tweeting to Health,” which used Fitbits, Twitter, and gamification to facilitate support for healthy lifestyle changes in overweight/obese (OW) and healthy weight (HW) young adults.	Twitter	Nonrandomized control trial	Steps in a day, eating habits, and a daily dietary.	OW participants had on average 11,222 daily steps versus 11,686 (HW). One-day challenges were successful in increasing steps. Participants increased fruit/vegetable intake (92%) and decreased their sugar-sweetened beverage intake (67%). Compliance with daily Fitbit wear (99% of all days OW vs. 73% HW) and daily dietary logging (82% OW vs. 73% HW) and satisfaction was high.
**Coccia C, Fernandes SM, and Altiti, J.** [43]	2020	Student between 18–24	USA	The main objective of this study was to determine the feasibility and efficacy of a social-media-based nutrition intervention using Twitter on nutrition knowledge, dietary practices, body mass index (BMI), self-efficacy, and social support among student-athletes.	Twitter	Experimental design	Eating habits, lifestyles, degree of interest and self-evaluation of eating habits	The results indicate a 6-week nutrition intervention delivered solely through social media resulted in increased nutrition knowledge (t = 22.23; *p* = 0.035), reduced fat intake (t = 21.57; *p* = 0.13), and decreased BMI (t = 2.32; *p* = 0.027) in student-athletes.
**Laranjo L, Quiroz JC, Tong HL, et al.** [44]	2020	Young adults between 19–35	Australia	This 6-month pilot study on a social networking mobile app connected to wireless weight and activity tracking devices had two main aims: to evaluate changes in BMI, weight, and physical activity levels in users from different BMI categories and to assess user perspectives on the intervention, particularly on social comparison and automated self-monitoring and feedback features.	A social networking mobile app	Experimental design	Evaluate changes in BMI, weight, and physical activity levels in users from different BMI categories.	There were no differences in BMI from baseline to postintervention (6 months) and between the two BMI groups. However, at 4 weeks, participants’ BMI decreased by 0.34 kg/m^2^ (*p* < 0.001), with a loss of 0.86 kg/m^2^ in the overweight-obese group (*p* = 0.01).
**Watanabe-Ito M, Kishi E, Shimizu Y** [45]	2020	Young adults between 19–22 years old	Japan	Evaluate the potential effectiveness of social media interactions with the use of dietary diaries on a smartphone app to motivate college students in raising self-awareness of their eating habits	Any social media platform	Online surveys	Eating habits, lifestyles, degree of interest and self-evaluation of eating habits	Participants gradually thought more about their eating habits from various perspectives when choosing a meal/drink, particularly with respect to maintaining well-balanced diets and introducing diverse ingredients. Participants evaluated their experiences as interesting/fun and reported familiarity with using the smartphone app and social media as the preferred method to keep track of their eating.
**Pope Z, Barr-Anderson D, Lewis B et al.** [46]	2019	Young adults between 18–30 years old	USA	Investigate the feasibility and initial effectiveness of an intervention combining Polar M400 smartwatch use and a twice-weekly based Facebook-delivered health education intervention on improving college students’ PA and dietary behaviors.	Facebook	Randomized controlled trial	BMI, eating habits (calories per day), self-efficacy, social support, intrinsic motivation	Participants implemented health education tips 1–3 times per week. We observed experimental and comparison groups to have 4.2- and 1.6-min/day increases in moderate-to-vigorous physical activity, respectively, at six weeks—partially maintained at 12 weeks. In both groups, similarly decreased body weight (experimental = −0.6 kg; comparison = −0.5 kg) and increased self-efficacy, social support, and intrinsic motivation were observed pre- and postintervention. Finally, we observed small decreases in daily caloric consumption over time (experimental = −41.0 calories; comparison = −143.3).
**Ruotsalainen H, Kyngas H, Tammelin T et al.** [47]	2015	Teenagers between 13–16 years old	Egypt	To assess the effects of a 12-week lifestyle guidance intervention delivered by Facebook, with or without self-control of physical activity, on physical activity and body mass index (BMI) in overweight and obese adolescents aged 13 to 16 years.	Facebook	Randomized controlled trial	Self-reported physical activity and screen time, BMI	The findings reported in this document suggest that a lifestyle guidance intervention delivered by Facebook aimed primarily at promoting physical activity among adolescents was not effective in increasing physical activity or decreasing BMI for study participants.
**Pagkalos I, Kokkinopoulou A, Weal M et al.** [48]	2017	Young adults between 16–31 years old	USA	Know the effectiveness of a SNApp through Facebook. It is called ‘NutriHeAl Activity Diary’.	Facebook	Randomized controlled trial	Physical activity, BMI	Facebook applications are a novel, customizable and powerful tool to collect all kinds of health data from the growing number of Facebook users.
**Ramalho S, Saint-Maurice P, Silva D et al.** [49]	2018	Teenagers between 13–18 years old	Germany	(1) Describe the study protocol of the randomized trial of controlled modification and (2) present descriptive reference information of the Portuguese sample.	Facebook	Descriptive study	BMI, body fat, health status, eating behavior, physical activity, psychological functioning	Depression, anxiety, stress, impulsivity and body fat percentage were inversely associated with health-related quality of life, while physical activity outside of school was positively related to the quality of life related to health. Compared to boys, girls showed statistically significant higher scores in psychological disorders, disturbed eating behaviors, impulsivity, were less active in school and obtained lower scores in health-related quality of life.
**Walker M, Thornton L, De Choudhury M et al.** [50]	2015	Young adults between 18–23 years old	USA	Expand current literature on associations between the use of Facebook and disordered eating behaviors in three ways.	Facebook	Randomized controlled trial	BMI, physical appearance, depression, anxiety, perfectionism, impulsiveness, self-efficacy	Our hypothesis is that higher Facebook intensity would be associated with higher disordered eating behavior. However, in the mediation analysis, we found that the association between the intensity of Facebook and the disorderly feeding was more complicated than we anticipated. Somehow, the intensity of Facebook, the emotional connection of an individual and the integration of the site in their daily lives, seems to be a double-edged sword for disorderly eating behavior.
**Merchant G, Weibel N, Patrick K et al.** [51]	2017	Young adults between 18–35 years old	Canada	Describe participants ‘exposure to a Facebook page designed to deliver content to overweight or obese college students in a randomized controlled trial of weight loss (*n* = 404) and examine participants’ commitment to behavior change campaigns to weight loss delivered through Facebook.	Facebook	Experimental design	BMI, social support, motivation, participation	Facebook can be used to offer evidence-based weight loss intervention content designed for university students, but the visible participation of participants is low, although there are numerous benefits of using Facebook such as the ability to iteratively adapt the content and provide support timely social and comments to participants among others.

## Abbreviations

BMI	Body mass index
PRISMA	Preferred Reporting Items for Systematic Reviews and Meta-Analysis
TYA	Teenagers and young adults

## Appendix A

### Search Terms

The complete search is detailed below:

(((((((((((((social media [Title/Abstract]) AND obesity [Title/Abstract]) OR social media [Title/Abstract]) AND overweight [Title/Abstract]) Social media OR [Title/Abstract]) AND bmi [Title/Abstract]) Social media OR [Title/Abstract]) AND body mass index [Title/Abstract])) OR (((((((((((((((((((social networks [title/abstract]) AND online [title/abstract]) AND obesity [title/abstract])) OR ((((social networks [title/abstract]) AND online [ title/abstract]) AND overweight [title/abstract])) OR ((((social networks [title/abstract]) AND internet [title/abstract]) AND obesity [title/abstract])) OR (((social networks [ title/abstract]) AND internet [title/abstract]) AND overweight [title/abstract])) OR (((social networks [title/abstract]) AND online [title/abstract]) AND bmi [title/abstract])) OR (((social networks [title/abstract]) AND online [title/abstract]) AND body mass index [title/abstract])) OR ((((social networks [title/abstract]) AND internet [title/abstract]) AND bmi [title/abstract])) OR (((social networks [title/abstract]) AND internet [title/abstract]) AND body mass index [title/abstract])) OR (((facebook [title/abstract]) AND obesity [title/abstract]))) OR ((((facebook [title/abstract]) AND overweight [title/abstract]))) OR ((((facebook [title/abstract]) AND bmi [title/abstract]))) OR ((((facebook [title/abstract]) AND body mass index [title/abstract]))) OR ((((twitter [title/abstract]) AND obesity [title/abstract]))) OR (((twitter [title/abstract]) AND overweight [title/abstract]))) OR (((twitter [title/abstract]) AND bmi [title/abstract]))) OR (((twitter [title/abstract]) AND body mass index [title/abstract]))))) OR (((((youtube [title/abstract]) AND obesity [title/abstract]))) OR (((youtube [ title/abstract]) AND overweight [title/abstract]))) OR ((((youtube [title/abstract]) AND bmi [title/abstract]))) OR ((((youtube [title/abstract]) AND body mass index [title/abstract])))).

**Table A1:** Scoring system for the methodological quality of the included studies.

Author(s)	Q1	Q2	Q3	Q4	Q5	Q6	Q7	Q8	Q9	Q10	Mean	Perc.	Value
Napolitano N, Hayes S, Bennett G et al. [38]	Good	Good	Good	Fair	Good	Poor	Poor	Good	Good	Poor	2.3	77%	High
Ling J, Robbins L, Zhang N et al. [39]	Good	Good	Good	Fair	Good	Poor	Fair	Good	Good	Poor	2.4	80%	High
Villiard H, Moreno M. [40]	Good	Good	Good	Good	Good	Can’t tell	Fair	Poor	Fair	Poor	2.1	70%	High
Krishnamohan S, Stalin P, Singh Z et al. [41]	Good	Poor	Good	Good	Fair	Poor	Poor	Good	Fair	Poor	2	67%	High
Chung AE, Skinner AC, Hasty SE et al. [42]	Good	Good	Good	Fair	Good	Good	Good	Good	Good	Good	2.9	86%	High
Coccia C, Fernandes SM, and Altiti, J. [43]	Good	Good	Good	Good	Good	Fair	Fair	Good	Fair	Good	2.7	80%	High
Laranjo L, Quiroz JC, Tong HL, et al. [44]	Good	Good	Fair	Good	Good	Fair	Good	Good	Good	Good	2.9	86%	High
Watanabe-Ito M, Kishi E, Shimizu Y [45]	Good	Good	Good	Good	Good	Fair	Fair	Good	Fair	Good	2.7	80%	High
Pope Z, Barr-Anderson D, Lewis B et al. [46]	Good	Good	Good	Good	Fair	Good	Fair	Good	Fair	Good	2.7	80%	High
Ruotsalainen H, Kyngas H, Tammelin T et al. [47]	Good	Good	Good	Poor	Good	Poor	Good	Good	Fair	Poor	2.3	77%	High
Pagkalos I, Kokkinopoulou A, Weal M et al. [48]	Poor	Can’t tell	Fair	Fair	Fair	Poor	Good	Good	Good	Fair	1.9	64%	Mod
Ramalho S, Saint-Maurice P, Silva D et al. [49]	Good	Good	Good	Poor	Fair	Fair	Poor	Good	Good	Poor	2.2	74%	High
Walker M, Thornton L, De Choudhury M et al. [50]	Good	Good	Good	Good	Good	Poor	Good	Good	Good	Poor	2.6	87%	High
Merchant G, Weibel N, Patrick K et al. [51]	Good	Good	Good	Good	Good	Can’t tell	Good	Good	Good	Can’t tell	2.4	80%	High

**Table A2:** Scoring system in Norman et al.

Author(s)	Q1	Q2	Q3	Q4	Q5	Q6	Q7	Q8	Q9	Perc.	Score	Value
Napolitano N, Hayes S, Bennett G et al. [38]	Yes	Yes	Yes	Yes	Yes	Yes	No	No	Yes	78%	7	High
Ling J, Robbins L, Zhang N et al. [39]	Yes	Yes	No	Yes	No	Yes	Yes	Yes	Yes	78%	7	High
Villiard H, Moreno M. [40]	No	No	No	No	No	Yes	No	No	Yes	22%	2	Low
Krishnamohan S, Stalin P, Singh Z et al. [41]	No	Yes	Yes	Yes	No	Yes	No	No	Yes	56%	5	Mod
Chung AE, Skinner AC, Hasty SE et al. [42]	Yes	Yes	Yes	Yes	Yes	Yes	Yes	No	Yes	89%	8	High
Coccia C, Fernandes SM, and Altiti, J. [43]	Yes	Yes	No	Yes	Yes	Yes	Yes	No	Yes	78%	7	High
Laranjo L, Quiroz JC, Tong HL, et al. [44]	Yes	Yes	No	Yes	No	Yes	No	Yes	Yes	67%	6	High
Watanabe-Ito M, Kishi E, Shimizu Y [45]	No	No	Yes	Yes	No	Yes	Yes	Yes	Yes	67%	6	High
Pope Z, Barr-Anderson D, Lewis B et al. [46]	Yes	Yes	No	Yes	No	Yes	No	No	Yes	56%	5	Mod
Ruotsalainen H, Kyngas H, Tammelin T et al. [47]	Yes	Yes	No	Yes	No	Yes	No	Yes	Yes	67%	6	High
Pagkalos I, Kokkinopoulou A, Weal M et al. [48]	Yes	Yes	Yes	Yes	No	Yes	No	No	Yes	67%	6	High
Ramalho S, Saint-Maurice P, Silva D et al. [49]	Yes	Yes	Yes	Yes	Yes	Yes	Yes	No	Yes	89%	8	High
Walker M, Thornton L, De Choudhury M et al. [50]	Yes	No	No	No	No	Yes	No	No	Yes	33%	3	Mod
Merchant G, Weibel N, Patrick K et al. [51]	Yes	Yes	No	Yes	Yes	Yes	Yes	No	Yes	78%	7	High
